# Neoadjuvant Chemotherapy Use in Bladder Cancer: A Survey of Current Practice and Opinions

**DOI:** 10.1155/2014/746298

**Published:** 2014-05-28

**Authors:** N. G. Cowan, Y. Chen, T. M. Downs, B. H. Bochner, A. B. Apolo, M. P. Porter, J. C. La Rochelle, C. L. Amling, T. M. Koppie

**Affiliations:** ^1^Department of Urology, Oregon Health and Science University, 3303 SW Bond Avenue, Portland, OR 97239, USA; ^2^Department of Public Health and Preventive Medicine, Oregon Health and Science University, 3303 SW Bond Avenue, Portland, OR 97239, USA; ^3^Department of Urology, University of Wisconsin, Madison, WI 53705, USA; ^4^MSKCC, Department of Surgery, Urology Service, New York, NY 10065, USA; ^5^National Cancer Institute, Medical Oncology Branch and Affiliates, Bethesda, MD 20892, USA; ^6^Department of Urology, University of Washington, Seattle, WA 98195, USA

## Abstract

*Objectives*. Level 1 evidence supports the use of neoadjuvant chemotherapy (NAC) to improve overall survival in muscle invasive bladder cancer; however utilization rates remain low. The aims of our study were to determine factors associated with NAC use, to more clearly define reasons for low utilization, and to determine the current rate of NAC use among urologic oncologists. *Materials and Methods*. Active members of the Society for Urologic Oncology were provided a 20-question survey. Descriptive statistical analysis was conducted for each question and univariate analysis was performed. *Results*. We achieved a response rate of 21%. Clinical T3/T4 disease was the most often selected reason for recommending NAC (87%). Concerns with recommending NAC were age and comorbidities (54%) followed by delay in surgery (35%). An association was identified between urologic oncologists who discussed NAC with >90% of their patients and medical oncologists “always” recommending NAC (*P* = 0.0009). NAC utilization rate was between 30 and 57%. *Conclusions*. Amongst this highly specialized group of respondents, clinical T3-T4 disease was the most common reason for implementation of NAC. Respondents who frequently discussed NAC were more likely to report their medical oncologist always recommending NAC. Reported NAC use was higher in this surveyed group (30–57%) compared with recently published rates.

## 1. Introduction

Approximately 73,000 new cases of bladder cancer are diagnosed each year in the USA with almost 15,000 people succumbing to the disease [1]. Five-year relative survival for stages II (T2a or T2b, N0, and M0) and III (T3a, T3b, or T4a, N0, and M0) bladder cancer remains poor at 63% and 46%, respectively [2]. Over the past two decades much work has been done to evaluate the role of chemotherapy in the treatment of muscle invasive bladder cancer (MIBC). In a prospective trial, Grossman et al. demonstrated a survival benefit using combination cisplatin-based neoadjuvant chemotherapy (NAC) for cT2-T 4aN0M0 bladder cancer [3]. A meta-analysis in 2005 of all published randomized trials to that point found an absolute 5-year overall survival benefit of 5% [4]. Subsequent studies including a 2011 international phase 3 trial update confirmed this finding [5]. Thus, there is level 1a evidence to support the use of neoadjuvant cisplatin-containing combination chemotherapy to improve overall survival in MIBC [6].

Despite the robust data supporting its use, there has been a relatively low rate of NAC utilization over the past decade, estimated to have increased from approximately 1% to 17% in the USA between 1998 and 2008 [7]. More recent USA data reports a 17% utilization rate at a high-volume tertiary center [8]. Reasons for this underutilization remain to be fully elucidated as few studies in the USA have broadly evaluated why patients are not receiving NAC. We sought to more clearly define these reasons and determine the frequency of NAC utilization by surveying a contemporary cohort of urologic oncologists.

## 2. Materials and Methods

The IRB approved survey was developed by the authors with the help from collaborators of the Bladder Cancer Think Tank and administered via an online survey tool. The 20-question survey consisted of questions examining physician training, practice patterns and setting, chemotherapy recommendation practices, and reasons why patients do or do not receive chemotherapy for muscle invasive bladder cancer for. Both neoadjuvant and adjuvant chemotherapy practice patterns were examined although the bulk of the questionnaire was aimed at elucidating practice patterns around neoadjuvant chemotherapy use. The survey consisted of questions in a variety of formats including the ability to free write in responses.

The survey was sent to all active members of the Society for Urologic Oncology in January 2012. We excluded respondents who identified themselves as medical oncologists or fellows.

At the time of survey administration there were 628 unique email addresses to which the survey was delivered. Of these, 241 were opened and 132 were completed for a response rate of 21%. Seven of the survey respondents did not meet inclusion criteria and therefore a total of 125 survey responses were analyzed. Descriptive statistical analysis was conducted for each survey question. A range of NAC utilization rates was determined by assuming respondents' selections were first at the upper then at the lower end of the provided response ranges. Weighted averages were then calculated at both the upper and the lower ends of ranges.

The association between two binary categorical variables was evaluated using the Chi-squared test. Logistic regression was used to assess the effect of potentially relevant factors on the categorical response variables that are of primary interest (i.e., whether the responders discussed NAC >90% of the time with their patients and whether >60% of their patients have received NAC prior to surgery). Statistical significance was defined as *P* < 0.05. All statistical analysis was conducted using SAS 9.3 (New York, Cary).

## 3. Results

Seventy percent of respondents considered their practice setting “university” while 23% selected “private practice.” Nearly 82% of physicians reported completing a urologic oncology fellowship. Of those surveyed, 66% perform >10 cystectomies per year and 19% perform between 8 and 10 per year. A dedicated GU medical oncologist was present at 81% of respondents' institutions. Regular GU multidisciplinary tumor board meetings were reported by 92% of respondents.

**Figure 1:**
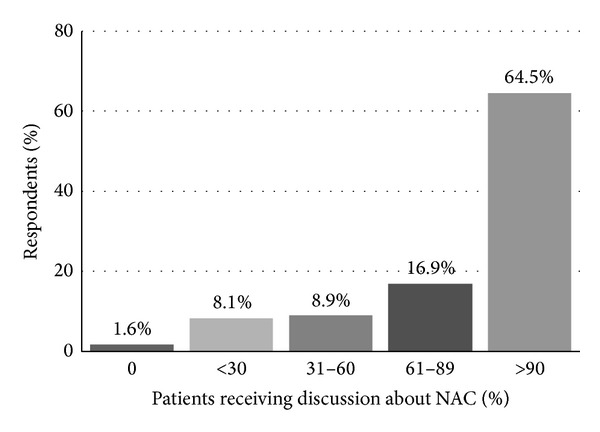
“When you counsel patients about radical cystectomy, with what proportion do you discuss NAC?”

**Figure 2:**
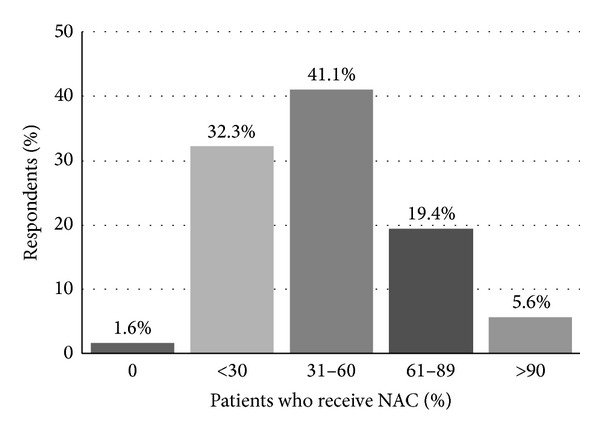
“What proportion of your patients undergoing radical cystectomy have received NAC?”

When asked how they incorporate NAC into their practice, 65% reported* discussing *NAC with a large majority (>90%) of their patients (Figure 1). Fifty-three percent reported* recommending* NAC for all eligible patients undergoing radical cystectomy (RC) for localized MIBC. However, only 41% of respondents noted that between one-third and two-thirds of their patients received NAC prior to RC (Figure 2). A utilization rate between 30 and 57% was calculated as described above.

**Figure 3:**
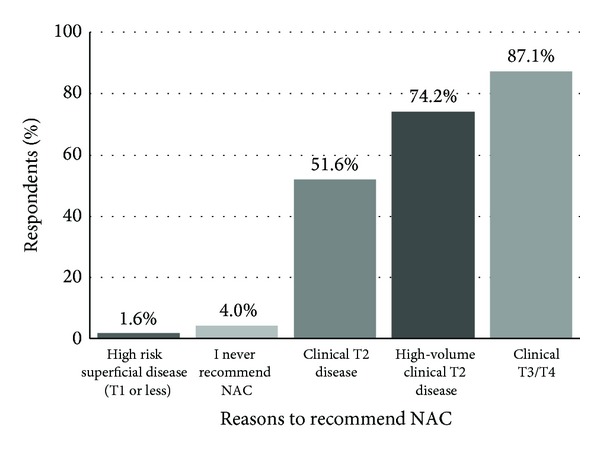
“When counseling a patient with localized bladder cancer, to which patients do you recommend NAC? (Select all that apply.)”

When asked their rationale for* recommending* NAC for localized bladder cancer, 87% indicated clinical T3/T4 followed by 74% who indicated high-volume clinical T2 disease (Figure 3).

**Figure 4:**
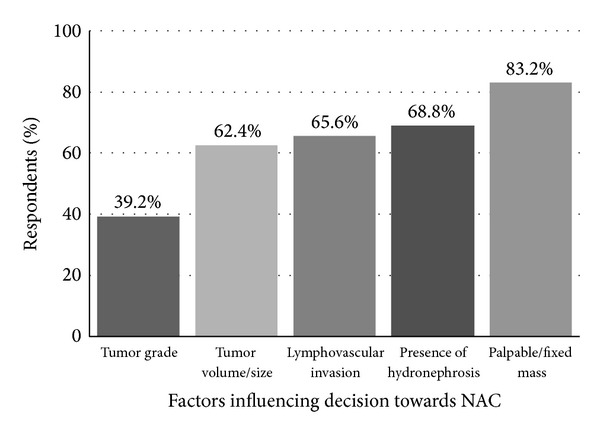
“Which of the following factors would influence your decision towards NAC for a patient with localized MIBC? (Select all that apply.)”

Interestingly, fewer respondents (51%) were* recommending* NAC for clinical T2 disease alone. When asked which factors influence their decision towards NAC for a patient with MIBC, 83% reported a palpable/fixed mass, 69% selected presence of hydronephrosis, 66% reported LVI, and 62% reported tumor size (Figure 4).

**Figure 5:**
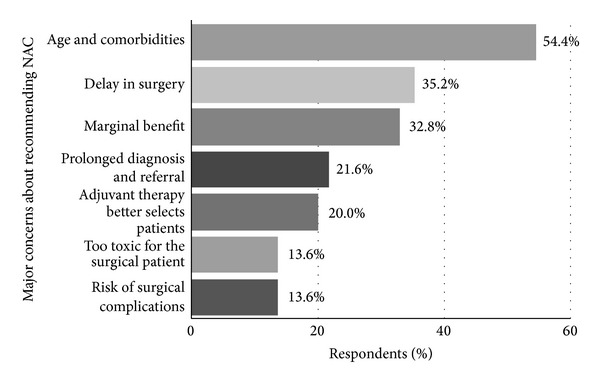
“What are your major concerns about recommending NAC? (Select all that apply.)”

The most frequently cited* concerns* about recommending NAC were age and comorbidities (54%) followed by delay in surgery (35%) and marginal benefit (33%) (Figure 5).

A smaller but significant portion of respondents wrote that they had no concerns with recommending NAC.

When asked to write the top reason why they* do not recommend* NAC for patients with MIBC, 50% reported medical risk (i.e., poor ECOG, comorbidities, poor renal function, age, and toxicity), 15% stated extent and type of disease, and 14% questioned the efficacy of NAC as their top reason. When asked the top reason that their patients* do not receive* NAC prior to RC, 48% wrote medical risk, 27% wrote patient choice, 6% said efficacy, and 5% said extent/type of disease.

When respondents were categorized into those who discuss NAC >90% of the time and those who discuss NAC <90% of the time, no association was found with practice setting, level of training, age, number of radical cystectomies performed, presence of a GU multidisciplinary meeting, or presence of a GU medical oncologist. A significant association was noted between those who discuss NAC >90% of the time and institutions where medical oncologists “always” recommend NAC (*P* = 0.0009). Respondents were then grouped based on reported rates of >60% or <60% of their patients having received NAC prior to surgery. Again no association was found with practice setting, level of training, age, number of radical cystectomies performed, presence of a GU multidisciplinary meeting, or presence of a GU medical oncologist. Physicians who discussed NAC >90% of the time were significantly more likely to report that >60% of their patients receive NAC (*P* < 0.0001).

## 4. Discussion

Muscle invasive bladder cancer continues to cause significant cancer specific mortality despite advances in surgical technique. After more than two decades of research on the use of chemotherapy in bladder cancer there now exists substantial evidence to support its use in the neoadjuvant setting [9]. In its 2011 update, the European Association of Urology gave a level 1a recommendation for the use of neoadjuvant cisplatin-containing combination chemotherapy in MIBC [6].

Despite the evidence supporting the use of NAC, clinicians in both the USA and Europe have been slow to incorporate it into the bladder cancer treatment paradigm, with the most recently published data reporting 12–17% of patients having received NAC [8, 10]. A number of previous studies have reported on NAC utilization from cancer databases and single institutions within the USA. In one of the first studies to investigate utilization rates, David et al. reviewed the National Cancer Data Base and found that NAC use was just over 1% between 1998 and 2003 [7]. A 2011 update of this database found NAC rates increasing from 6 to 13% between 2003 and 2007 [11]. A more contemporary retrospective review by Raj et al. looked at a single institution series from a high-volume tertiary care center [8]. They found that 17% of their patients with MIBC received cisplatin-based NAC. Contemporary European data suggests a similar pattern. Results from a 2012 survey of NAC use amongst 133 major European centers also found low utilization rates, with roughly 12% of MIBC patients being considered for NAC [10].

The goal of our study was to evaluate the rate and rationales for utilization of NAC prior to RC among experts in urologic oncology. Respondents in our study were largely fellowship trained urologic oncologists with relatively high-volume cystectomy practices, and thus our study provides unique insights into practice patterns and preferences among thought leaders in urologic oncology. Our survey sample was a relatively homogeneous population. A large majority (71.6%) of our respondents were from academic facilities although a solid minority was from private practice (22%). Nearly 79% of physicians reported completing a urologic oncology fellowship and a majority (63.6%) were performing >10 cystectomies per year. These numbers clearly point to the fact that these physicians have a unique interest in urologic oncology and in particular bladder cancer.

Our study demonstrates that even among experts in our field, NAC for bladder cancer is infrequently utilized (30–57%), despite the fact that over 90% discuss the option of NAC to candidate patients. Our study further elucidates the rationale for this disparity. Most urologic oncologists we surveyed hesitated to recommend chemotherapy due to concerns about age and comorbidities, delay in surgery, and a perceived marginal therapeutic benefit. These concerns are consistent with those previously reported in both the USA and Europe [10, 12].

NAC utilization patterns within our surveyed cohort appear biased in favor of treating patients with advanced clinical features. While level 1a evidence demonstrates a survival benefit for all patients with T2 or greater disease, some have questioned the quality of the data as it pertains to T2 disease, citing concerns with understaging and unfavorable overall survival rates when compared to contemporary RC only series [13]. Preference towards treatment of more aggressive disease was apparent in our results as only half of respondents cited clinical T2 disease (51%) as the most common reason for recommending NAC. Both clinical T3/T4 (87%) and high-volume clinical T2 (74%) were more frequent reasons for recommending NAC. Other factors influencing our respondents' choice of NAC were the presence of a palpable/fixed mass and hydronephrosis, both markers of more aggressive disease. Notably, few of those surveyed by our study (12%) had a major concern about the quality of clinical trial data that support NAC prior to RC.

Explanations have been proposed to rationalize the low rates of NAC utilization, yet only few contemporary retrospective series have been published on this topic. Reasons commonly cited include concerns about delay in surgery, patient preferences, perceived marginal benefit, and concerns about morbidity resulting from chemotherapy [8, 12, 14]. While the adverse effects of cisplatin-based chemotherapy are well known (myelosuppression, gastrointestinal side effects, and nephrotoxicity) these do not appear to prevent patients from moving on to subsequent RC. Data from SWOG 8710 demonstrated that the RC rates did not differ between the treatment and the control arms. Eighty-two percent of patients in the NAC + RC arm completed RC, versus 81% of patients in the RC alone arm. The proportion of patients who did not undergo RC for medical reasons was also similar between the two groups. Fifty-nine percent (16/27) of the NAC + RC group did not complete RC for medical reasons, versus 66% (20/30) in the RC alone group. Furthermore, other studies demonstrate that most patients who undergo NAC are able to complete their therapy. In the largest ever randomized trial of NAC for MIBC, the International Collaboration of Trialists group reported that 79% of patients assigned to receive NAC completed all three cycles and only 4 patients (1.4%) did not receive their planned RC due to toxic effects from NAC [15]. It is important to note that these data are from large clinical trials and may not be reflective of routine clinical practice.

Delay in surgery has also been cited as a concern of NAC and is supported by studies showing that RC delay >90 days after diagnosis of MIBC adversely affects pathologic stage and survival outcomes in patients who undergo primary surgery [16, 17]. Interestingly, this concern may not be justified in patients selected to receive NAC as shown in a 2012 study by Alva et al. [18]. These authors concluded that cystectomies performed within 10 weeks after NAC did not compromise patient survival. They noted that procedural scheduling was the most common reason for RC delivery beyond 10 weeks and occurred disproportionately in those receiving NAC at an outside institution, confirming the need for good communication between the surgical and medical teams.

Renal toxicity is a known complication of cisplatin-based chemotherapy, particularly among patients with baseline renal insufficiency. Raj et al. examined renal function among 238 patients who underwent RC for bladder cancer and found that 70% of patients with stage T2 disease were eligible for cisplatin-based chemotherapy prior to surgery. This highlights that a large majority of patients are candidates for NAC based on renal function. Despite this data, our results suggest that renal toxicity remains a significant concern in recommending NAC to patients. In the adjuvant setting renal insufficiency remains a concern, yet adjuvant chemotherapy is preferred by many urologists because postoperative treatment relies on more accurate pathologic staging from the RC specimen. Studies have demonstrated that one-quarter to one-half of patients have chronic kidney disease (estimated GFR <50 mL/min/1.73 m^2^) that precludes administration of chemotherapy after RC [19, 20]. The proportion of eligible patients is therefore similar to the amount that is eligible in the neoadjuvant setting. Data on chemotherapy use in the adjuvant setting also suggests lower rates of completion. A single institution series by Eldefrawy et al. found that only 35.5% of patients completed the prescribed number of cycles, with hematologic complications being the most frequent reason for noncompletion [21].

NAC utilization rates appear to be higher among the urologic oncologists surveyed in this study when compared to previously reported rates from urologists in general. This difference may be due to the fact that urologic oncologists are frequently engaged in multidisciplinary treatment collaborations which impact their practice patterns. Our univariate analysis demonstrates that NAC utilization is high among urologic oncologists who frequently (>90%) discuss NAC with candidate patients and that NAC is more frequently discussed by urologic oncologists from institutions where medical oncologists strongly support NAC prior to RC. These data suggest that improved collaboration between medical oncologists and urologists might lead to increased NAC utilization. Improved patient counseling may also play a role, as our study demonstrates that only one-quarter of patients (27%) elect not to undergo NAC when given the option.

This study does have weaknesses. We did not specify the exact NAC regimen (e.g., cisplatin versus carboplatin) in any of the survey questions and therefore this may confound the results. Recall bias is a major concern with survey studies and may account for differences in our data compared to nonsurvey style studies. Additionally, our survey sample was a relatively homogenous population of physicians whose training and interest in urology are focused on the care of oncologic diseases. This may have limited our ability to find associations between survey responses and also prevent the data from being generalized to the larger urologic community. Lastly, with a response rate of 21%, selection bias within our survey sample may be present.

## 5. Conclusions

Clinical T3-T4 disease was the most commonly reported reason for recommending NAC followed by high-volume T2 disease. Age and comorbidities followed by delay in surgery were the top concerns impacting the recommendation for and use of NAC. Respondents who frequently discussed NAC were more likely to report their medical oncologist always recommending NAC. Self-reported NAC use appears to be higher in this group of urologic oncologists (30–57%) compared to data on utilization rates reported recently from a broader urologist population.

## Supplementary Material

A replica of the survey submitted to members of the Society for Urologic Oncology for this study is available in Supplementary Material for review.
