# Supplementary material for: Neoadjuvant Chemotherapy Use in Bladder Cancer: A Survey of Current Practice and Opinions
Source: Adv Urol. 2014 May 28;2014:746298. doi: 10.1155/2014/746298 (PMC4058463; doi:10.1155/2014/746298)
Supplement: Supplementary file 1 — A replica of the survey submitted to members of the Society for Urologic Oncology for this study is available in Supplementary Material for review. [file 746298.f1.pdf]

1. Neoadjuvant and adjuvant chemotherapy for T2-T4N0M0 bladder cancer

1. What is your practice setting?

- ☐ University
- ☐ Government
- ☐ Veteran's Administration
- ☐ Other non-profit
- ☐ Private practice

2. Select the response that describes your training:

- ○ Urology residency
- ○ Fellowship training (non-oncology)
- ○ Urologic oncology fellowship
- ○ Other

Other (please specify)

3. What is your age?

- ☐ <40 years
- ☐ 40-50 years
- ☐ 50-60 years
- ☐ 60-70 years
- ☐ >70 years

4. Approximately how many radical cystectomies do you perform per year?

- ○ <1
- ○ 1-3
- ○ 4-7
- ○ 8-10
- ○ >10

5. Do you have a dedicated GU medical oncologist at your institution?

- ○ Yes
- ○ No

6. Do you have regular GU multidisciplinary tumor board meetings/rounds at your institution?

- Yes
- No

7. Do the medical oncologists at your institution RECOMMEND NEOADJUVANT chemotherapy for muscle invasive bladder cancer?

- Yes
- No
- The medical oncologists at my institution are ambivalent

8. Which statement most closely characterizes how you incorporate NEOADJUVANT chemotherapy into your practice? (Select all that apply)

- [ ] I do not recommend neoadjuvant chemotherapy
- [ ] I DISCUSS neoadjuvant chemotherapy with all patients for whom radical cystectomy is an option.
- [ ] I RECOMMEND neoadjuvant chemotherapy for all ELIGIBLE patients undergoing radical cystectomy for localized muscle invasive bladder cancer (T2-4, N0, M0).
- [ ] I use neoadjuvant chemotherapy to downstage locally advanced disease prior to cystectomy.
- [ ] I use neoadjuvant chemotherapy to select patients with metastatic disease who may be candidates for consolidative cystectomy.

9. When you counsel patients about radical cystectomy, with what proportion do you DISCUSS NEOADJUVANT chemotherapy?

- 0
- <30%
- 31-60%
- 61-89%
- >90%

10. What proportion of your patients undergoing radical cystectomy HAVE RECEIVED NEOADJUVANT chemotherapy prior to surgery?

- 0
- <30%
- 31-60%
- 61-89%
- >90%

11. When counseling a patient with localized bladder cancer, to which patients do you RECOMMEND NEOADJUVANT chemotherapy? (Select all that apply)

- ☐ I never recommend neoadjuvant chemotherapy
- ☐ High risk superficial disease (T1 or less)
- ☐ Clinical T2 disease
- ☐ Clinical T3/T4

Other (please specify)

12. Which of the following factors would influence your decision towards NEOADJUVANT chemotherapy for a patient with localized muscle invasive bladder cancer? (Select all that apply)

- ☐ Lymphovascular invasion
- ☐ Carcinoma on situ
- ☐ Multifocality
- ☐ Tumor volume/size
- ☐ Tumor grade
- ☐ Presence of hydronephrosis
- ☐ Palpable/fixed mass (T3 disease)
- ☐ None of the above

*13. What are your major concerns about RECOMMENDING NEOADJUVANT chemotherapy? (Select all that apply)

- ☐ Preoperative chemotherapy is too toxic for the surgical patient
- ☐ Risk of surgical complications after chemotherapy
- ☐ Age and comorbidity of patients undergoing cystectomy in my practice
- ☐ Improvement from neoadjuvant chemotherapy is marginal
- ☐ Clinical trials to date flawed: no surgical controls
- ☐ Clinical trials to date flawed: TUR staging inaccuracies.
- ☐ Neoadjuvant chemotherapy delays surgery
- ☐ Patients referred to me are already delayed due to prolonged diagnosis and referral process
- ☐ Patients are referred to me for cystectomy, not chemotherapy
- ☐ Adjuvant therapy selects patients more appropriately for treatment
- ☐ Adjuvant therapy is better than neoadjuvant therapy
- ☐ Medical oncologist overburdened
- ☐ Medical oncologist doesn't recommend neoadjuvant chemotherapy
- ☐ Process takes too long, process with medical oncology not coordinated.
- ☐ Medical oncologists in my community administer non-standard chemotherapy (eg carboplatin)

Other (please specify)

____

*14. What are the TOP reasons that you do NOT RECOMMEND NEOADJUVANT chemotherapy for patients with muscle invasive disease.

One ______________________________

Two ______________________________

Three ______________________________

15. What are your TOP reasons that your patients do NOT RECEIVE NEOADJUVANT chemotherapy prior to cystectomy for muscle invasive bladder cancer?

One ______________________________

Two ______________________________

Three ______________________________

16. When counseling cystectomy candidates, what proportion of patients do you discuss ADJUVANT chemotherapy?

- ○ 0
- ○ <30%
- ○ 31-60%
- ○ 61-89%
- ○ >90%

17. When faced with a post-cystectomy patient who had no evidence of metastatic disease preoperatively, to whom do you recommend ADJUVANT chemotherapy? (Select all that apply)

- ☐ I never recommend adjuvant chemotherapy
- ☐ CIS
- ☐ Lymphovascular invasion
- ☐ pT3 disease
- ☐ pT4 disease
- ☐ N1 disease
- ☐ N2 disease
- ☐ N3 disease
- ☐ Positive surgical margin
- ☐ Grossly unresected disease
- ☐ Other

Other (please specify)

____

18. What are your major concerns about administering ADJUVANT chemotherapy?

- Adjuvant chemotherapy trials to date have been underpowered
- Comorbidity of patients after cystectomy
- Delays patient recovery
- Risk of overtreatment
- Prefer to wait for evidence of metastatic disease
- Patient preference
- Medical oncologist preference
- Medical oncologist opinion about efficacy of adjuvant treatment
- Other

Other (please specify)

19. Do you currently administer NEOADJUVANT chemotherapy prior to nephroureterectomy for upper tract urothelial carcinoma?

- Yes
- No
- Other

Other (please specify)

20. Would you refer patients for NEOADJUVANT chemotherapy for urothelial carcinoma of the upper tract if there was appropriate data supporting its use?

- Yes
- No
- Other

Other (please specify)
